# Targeted inhibition of ERα signaling and PIP5K1α/Akt pathways in castration‐resistant prostate cancer

**DOI:** 10.1002/1878-0261.12873

**Published:** 2020-12-16

**Authors:** Julius Semenas, Tianyan Wang, Azharuddin Sajid Syed Khaja, AKM Firoj Mahmud, Athanasios Simoulis, Thomas Grundström, Maria Fällman, Jenny L. Persson

**Affiliations:** ^1^ Department of Molecular Biology Umeå University Sweden; ^2^ Department of Clinical Pathology and Cytology Skåne University Hospital Malmö Sweden; ^3^ Division of Experimental Cancer Research Department of Translational Medicine Lund University Clinical Research Centre in Malmö Sweden; ^4^ Department of Biomedical Science Malmö University Sweden

**Keywords:** castration‐resistant prostate cancer, estrogen receptor, PI3K/AKT pathway and tamoxifen, PIP5K1α, targeted therapy

## Abstract

Selective ERα modulator, tamoxifen, is well tolerated in a heavily pretreated castration‐resistant prostate cancer (PCa) patient cohort. However, its targeted gene network and whether expression of intratumor ERα due to androgen deprivation therapy (ADT) may play a role in PCa progression is unknown. In this study, we examined the inhibitory effect of tamoxifen on castration‐resistant PCa *in vitro* and *in vivo*. We found that tamoxifen is a potent compound that induced a high degree of apoptosis and significantly suppressed growth of xenograft tumors in mice, at a degree comparable to ISA‐2011B, an inhibitor of PIP5K1α that acts upstream of PI3K/AKT survival signaling pathway. Moreover, depletion of tumor‐associated macrophages using clodronate in combination with tamoxifen increased inhibitory effect of tamoxifen on aggressive prostate tumors. We showed that both tamoxifen and ISA‐2011B exert their on‐target effects on prostate cancer cells by targeting cyclin D1 and PIP5K1α/AKT network and the interlinked estrogen signaling. Combination treatment using tamoxifen together with ISA‐2011B resulted in tumor regression and had superior inhibitory effect compared with that of tamoxifen or ISA‐2011B alone. We have identified sets of genes that are specifically targeted by tamoxifen, ISA‐2011B or combination of both agents by RNA‐seq. We discovered that alterations in unique gene signatures, in particular estrogen‐related marker genes are associated with poor patient disease‐free survival. We further showed that ERα interacted with PIP5K1α through formation of protein complexes in the nucleus, suggesting a functional link. Our finding is the first to suggest a new therapeutic potential to inhibit or utilize the mechanisms related to ERα, PIP5K1α/AKT network, and MMP9/VEGF signaling axis, providing a strategy to treat castration‐resistant ER‐positive subtype of prostate cancer tumors with metastatic potential.

## Graphical abstract

In the present study, we unrevealed the effect of tamoxifen on ERα and PIP5K1α/AKT that are responsible for prostate cancer growth and invasion. Tamoxifen in combination treatment with PIP5K1α inhibitor, ISA‐2011B, led to tumor regression in xenograft mice. We found that depletion of tumor‐associated macrophages using clodronate sensitized the inhibitory effect of tamoxifen on prostate cancer.

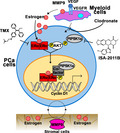

**Abbreviations**
CRPC: castration‐resistant prostate cancer
DHT: dihydrotestosterone
E2: estradiol
ERα: estrogen receptor alpha
GO: gene ontology
NG‐CHM: Next‐Generation Clustered Heatmaps
PCa: prostate cancer
TMAs: tissue microarrays

## 1 Introduction

Treatment options for metastatic prostate cancer (PCa) remain limited. PCa often inevitably progressed to castration‐resistant disease after initial androgen deprivation therapy. Thus, using agents to target androgen and its receptor AR lack the effectivity for castration‐resistant PCa and render the tumor to develop resistance to anti‐androgen‐related therapies [1, 2]. It is an urgent need to develop new treatment strategies to effectively target subtypes of PCa which do not respond to anti‐androgen therapies.

Estrogen agonist/antagonist, tamoxifen (1‐[*p*‐dimethylaminoethoxyphenyl]‐1,2‐diphenyl‐1‐butene) has been shown to inhibit growth of PCa cells in a few preclinical studies [3]. Clinically, high‐dose tamoxifen therapy was well tolerated in a heavily pretreated patient cohort with hormone‐refractory prostate cancer as reported in clinical trial studies [4]. However, the precise mode of action and specific targeted network factors by tamoxifen in primary tumors of PCa patients or in PCa cell lines have not been reported. Tamoxifen is the gold standard for the adjuvant treatment of breast cancer in pre‐ and postmenopausal patients with estrogen receptor alpha (ERα)‐positive tumors and is the first selective estrogen receptor modulator [5, 6]. Tamoxifen exerts its effect either by disrupting estrogen biosynthesis or inhibiting ERα activity [7]. However, for patients with breast cancer or PCa who suffer bone metastasis, alternative tailed treatment to eradicate metastatic lesions in the skeleton of patients with bone metastasis are needed [8]. Thus, understanding of the mechanisms that underlying targets of tamoxifen in PCa is essential for development of new therapy.

Estrogen (*17β‐estradiol*, E2) exerts its effects by binding to specific estrogen receptors, and it is converted from androgen by aromatase that is dramatically increased in metastatic PCa tissue compared with primary tumors [9, 10, 11]. Expression of ERα has been shown to be abnormally increased in castration‐resistant PCa (CRPC) and metastatic tumors [12, 13]. ERα‐mediated signaling can be broadly classified as either ligand‐dependent or ligand‐independent which trigger distinct signaling pathways to regulate a broad range of estrogen responsive genes [9, 11, 14, 15, 16]. ERα‐induced estrogen effects were suggested to promote epithelial versus mesenchymal transition (EMT) by regulating the activity of NOTCH1 signaling pathway. Further, E2 promoted tumor formation and metastasis, which were inhibited by tamoxifen treatment in mouse models [17]. It has also been reported that estrogen/ ERα axis plays an important role in promoting EMT‐associated events during PCa development [18].

PIP5K1α is a lipid kinase similar to PI3K [19]. PIP5K1α acts upstream of PI3K/Akt by producing PIP_2_, a major lipid substrate for triggering PI3K activity. PI3K converts PIP_2_ into PIP_3_, a key molecule for activation of Akt family of serine/threonine kinases [20]. PIP5K1α promotes PCa progression by regulating PI3K/Akt and AR pathways [21]. Further, PIP5K1α acts on PI3K/Akt and ERα pathways in promoting ERα‐positive breast cancer invasion. Silencing of PIP5K1α sensitizes breast cancer cells to tamoxifen treatment, which in turns blocks ERα signaling [22].

We have developed ISA‐2011B, a novel selective inhibitor of PIP5K1α, that exhibits specific inhibitory effect on advanced PCa by inhibiting elevated levels of PI3K/Akt and AR [21, 23, 24]. As PIP5K1α acts upstream of PI3K/Akt/PTEN and AR, a better understanding of the consequences of inhibiting these pathways and its effects on estrogen signaling pathways will have a significant impact on refining therapeutic strategies and improving clinical outcome. It is therefore of great interests to investigate whether tamoxifen alone or in combination with ISA‐2011B may be used as effective treatment strategies for targeted therapy for subgroups of PCa.

## 2 Materials and Methods

### 2.1 Patient cohort, tissue specimens, and tissue microarrays

Paraffin‐embedded tissues including benign prostate hyperplasia (BPH) (*n* = 96) and primary PCa (*n* = 113), and metastatic lesions in lymph nodes (LN) (*n* = 10), bone (*n* = 16), and lung (*n* = 4) were used to generate tissue microarrays (TMAs). The study was approved by the Ethics Committees, and the Helsinki Declaration of Human Rights was strictly observed.

### 2.2 The Cancer Genome Atlas (TCGA) program

Raw RNA‐seq counts and relevant clinical data for TCGA prostate adenocarcinoma (TCGA‐PRAD) provisional cohort of 498 prostate cancer patients were extracted from Genomic Data Commons (GDC) data portal. In addition to that, clustered heatmaps of normalized RNA‐seq counts along with selected RNA expression covariate data were acquired and adapted from Next‐Generation Clustered Heatmaps (NG‐CHM) viewer (software version: 2.14.2, map version: 1.1.0). Clustered data was established using iCluster package that uses multiple genomic data types (e.g., copy number, gene expression, DNA methylation) in order to fit a regularized latent variable model based clustering that generates an integrated cluster assignment based on joint inference across data types. A total of 1 μg of RNA/sample was used for library preparation, and RNA sequencing was performed using Illumina PE150 sequencer (Novegene, Beijing, China).

### 2.3 Mapping and bioinformatics analysis

Clean PE150 reads were mapped on human reference genome, GRCh38.p12, acquired from Gencode, using hisat2 (V2.1.0) aligner. Mapped reads were then sorted and indexed using samtools (v1.9) and counted using htseq (v0.9.1). All bioinformatics analysis of raw read counts were performed on R (v3.5.1), using different gene expression analysis package, DESeq2 (v1.22.2). Gene set enrichment analysis of biological process on gene ontology was performed using the Gene Ontology (GO) stats R package, and the conditional parameter of preranked gene lists was performed using GSEA (v4.0.3) [25]. This was done to identify pathways that were more represented in the set of significantly differentially expressed genes than would be expected by chance alone. All *P* values were also adjusted for the fact that many genes were being simultaneously analyzed by controlling the false discovery rate (FDR) with the Benjamini–Hochberg method [26, 27, 28, 29]. Enrichment maps using enrichment phenotypes with false discovery rate (FDR) cutoff of 0.1, *P* value cutoff of 0.005, and overlap coefficient of 0.5 were visualized using Enrichment Map (v3.3.0) on Cytoscape (v3.8.0) [30, 31].

### 2.4 Immunohistochemistry

Immunohistochemistry on tumor tissue arrays was performed as previously described [32]. The staining procedure was performed using a semiautomatic staining machine (Ventana ES; Ventana Inc.). The staining intensity was scored as 0 (negative), 1 (weakly positive or positive), 2 (moderate positive), or 3 (strongly or very strongly positive).

#### 2.4.1 Cell culturing and treatments

VCaP (RRID:CVCL_2235), PC‐3 (RRID:CVCL_0035) were purchased from American Type Culture Collection (ATCC, Manassas, VA, USA). All experiments were performed with mycoplasma‐free cells. For treatment with 17‐β estradiol, the increased doses were used. For treatment with dihydrotestosterone (DHT), 10 nm DHT was used. For treatment experiments in using 17‐β estradiol, phenol‐red free medium containing 10% charcoal‐stripped serum was used. Vehicle control 0.1% Dimethyl sulfoxide (DMSO, Sigma Aldrich, Stockholm, Sweden) was used. For treatment with tamoxifen at 20 µm was used. PIP5K1 alpha inhibitor: ISA‐2011B, a diketopiperazine fused C‐1 indol‐3‐yl substituted 1,2,3,4‐tetrahydroisoquinoline derivative [21], at a final concentration of 50 µm in 0.1% DMSO was used for treatment for 48 h.

### 2.5 Proliferation assay

The effects of *17β*‐*Estradiol* on PCa cell lines were determined using the nonradioactive MTS proliferation assay (Promega Biotech) and/or Resazurin (R&D Systems, Abingdon, UK) assays were used according to the manufacturer’s protocol. The detailed information is described in the Supporting information Appendix S1.

### 2.6 Mouse models of xenograft PCa tumors and treatment

The animal studies were approved by the Swedish Regional Ethical Animal Welfare Committee. The animal welfare and guidelines were strictly followed. Athymic NMRI nude male mice (Charles River Biotechnology, MA, USA) aged 6 weeks and weighing 25–29 g were used. 1 × 10^6^ PC‐3 cells/mouse were subcutaneously implanted into mice. After tumor were established, tumor diameters were measured using calipers, and volumes were calculated using the equation (a × b^2^/2), where a and b represent the larger and smaller diameters, respectively. Mice were randomized and were treated with vehicle (control), tamoxifen (40 mg/kg), ISA‐2011B (40 mg/kg) or combinations of tamoxifen (20 mg/kg), and ISA‐2011B (20 mg/kg) by intraperitoneal injection every second day (*n* = 5 mice/group).

### 2.7 Mouse models for macrophage‐depletion and tumor spheroid formation assays

Subcutaneously implanted PC‐3 tumors were grown into approximately 400 mm^3^ and were randomized into 6 groups (3–4 mice per group). Mice from vehicle control, Tamoxifen, and ISA‐2011B treatment groups were randomized and administered either Encapsome^®^ or Clodrosome^®^. Mice were treated with Encapsome® or Clodronate via intratumoral injection in between the treatments with vehicle control, Tamoxifen, or ISA‐2011B, which was administered intraperitoneally. For xenograft tumor‐derived spheroid formation assays, 5 × 10^3^ single cell suspension were cultured in suspension in modified tumor spheroid semi‐solid medium for 20 days and then counted.

### 2.8 Immunoblot, Immunoprecipitation analysis, and subcellular fractionation

Subcellular fractionation, immunoblot, and immunoprecipitation analysis were performed as described previously [21]. For immunoprecipitation analysis, antibody against ERα was used to pull down the immune complexes, and antibody to IgG (Thermo Fisher Scientific, Sweden) was used as a negative control.

### 2.9 Immunofluorescence analysis

The PCa cells were seeded on glass coverslips and were subsequently treated with the different agents. Cells were fixed with 4% paraformaldehyde in PBS. The images were viewed and taken under an Olympus AX70 fluorescent microscope and software ACT2U (ACT2U version. 1.5, Stockholm, Sweden) was used as described in [21].

### 2.10 Induced overexpression via transfections

Transfections were performed using TransIT‐X2 transfection reagent, according to the manufacturer’s protocol (Mirus Bio LLC, Madison, WI, USA). For transient or stable transfection, control vector containing full‐length human ERα cDNA or empty control vector pLX‐304 were used. Cells overexpressing ERα or control vector were transient transfected into PC‐3 cells. The phenol‐red free medium containing 10% charcoal‐stripped serum was used for culturing the cells. All plasmid vectors were purchased from PlasmID Repository, Harvard Medical School.

### 2.11 Flow cytometry (FACS)‐based cell cycle analysis and apoptosis assay

Apoptosis of breast cancer cells after treatment with ISA‐2011B were performed using FACS‐based assays. FITC or PE‐conjugated Annexin V and 7‐AAD was used for apoptosis assay according to the manufacturers’ Protocol (BD Biosciences, San Jose, CA, USA). Data was analyzed using FCS Express (DeNovo Software, CA, USA), flowjo (Tree Star, Inc., OR, USA), and CytExpert (Beckman Coulter, FL, USA) softwares.

### 2.12 Holomonitor analysis

The digital Holomonitor M4 live cell imaging system and softwares were applied for measuring cell morphological changes and motility in response to drug treatment (PHI technologies, Lund Sweden). The microscope is placed in incubator at 37 °C and 5% CO_2._ The images of live cells were captured every 10 min during the entire treatment period of 48 h.

### 2.13 Statistical analysis

Tukey test, *t*‐test, Kruskal–Wallis/ANOVA, and Spearman rank correlation tests were performed. The immunohistochemistry H‐scores were log‐transformed to overcome the non‐normal distribution of H score data. The mean is the average value of all samples. The standard deviation (SD) is an indication of variability of all samples. The precision of the sample mean is indicated by standard error. Confidence levels are expressed using 95% confidence interval (CI). All statistical testes were two‐sided, and *P* values less than 0.05 were considered to be statistically significant. Data presented are representative of at least three independent experiments. Statistical software, Social Sciences software (SPSS, version 21, Chicago, IL, USA), was used. For *in vitro* experiments, at least three independent experiments were performed. Detailed statistical calculations were described in relevant figure text.

## 3 Results

### 3.1 The effects of tamoxifen on estrogen signaling pathway in PC‐3 cells

**Fig. 1:**
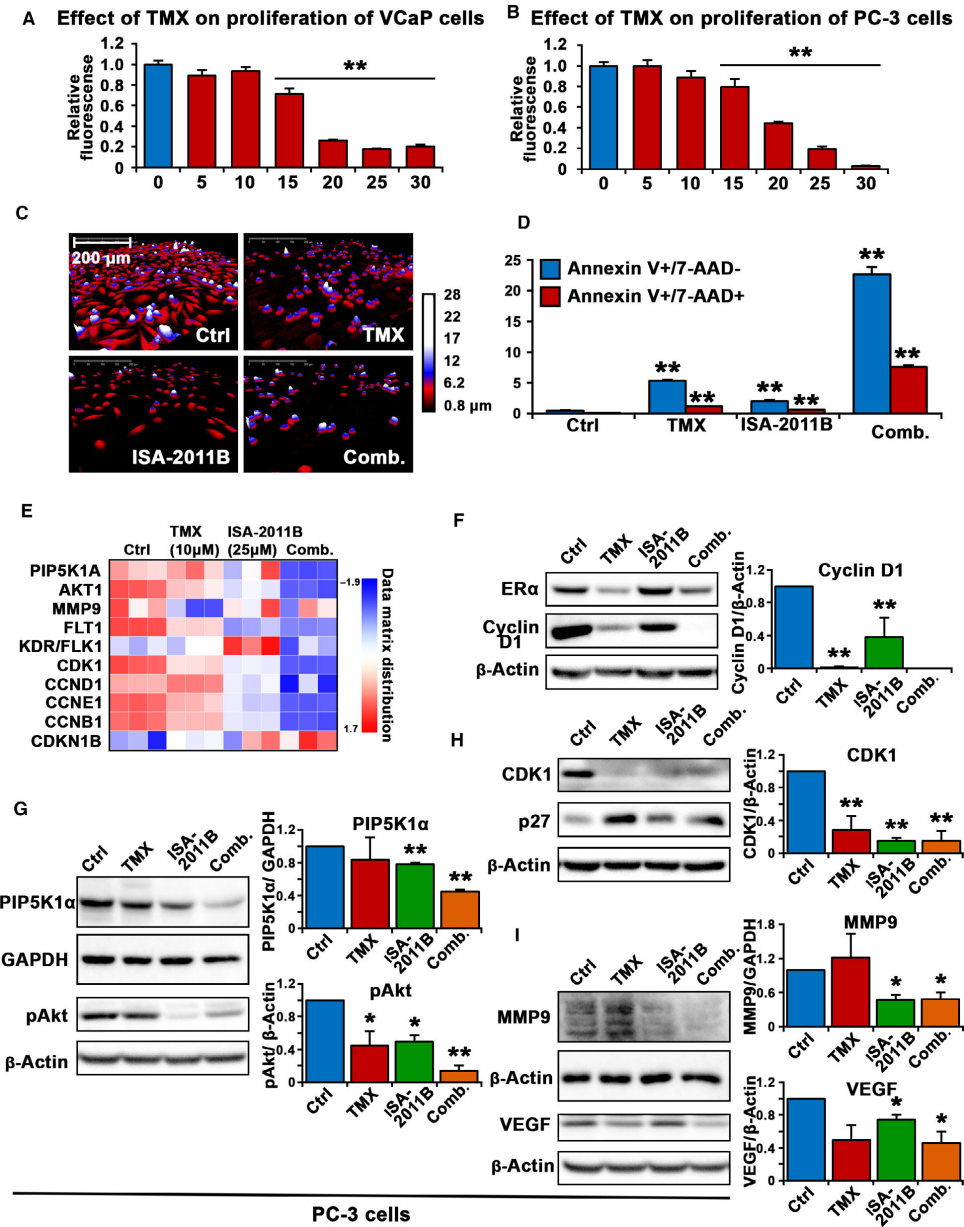
The inhibitory effect of tamoxifen and its downstream targets in PCa cells. (A and B). Dose‐dependent inhibitions of tamoxifen (TMX) on the proliferation of VCaP cells and PC‐3 cells are shown. TMX at 5, 10, 15, 20, 25 and 30 µm was used. Data is presented as average of three independent experiments (±SD). Student’s *t*‐test was used to compare the treatment groups. *P < *0.05, as indicated by “*”, *P* < 0.01, as indicated by “**”. (C). The Holomonitor M4 live cell imaging system was applied to capture PC‐3 cells in culture during the entire treatment period. Representative microphotographs of PC‐3 cells treated with vehicle control (Ctrl), tamoxifen (TMX), ISA‐2011B (ISA‐2011B), or combination of TMX and ISA‐2011B (Comb.) for 48 h are shown. Scale bar of 200 μm is indicated. (D). The effects of vehicle control (Ctrl), tamoxifen (TMX), ISA‐2011B (ISA‐2011B), or combination of TMX and ISA‐2011B (Comb.) were determined by using was determined using annexin‐7AAD‐based flow cytometry analysis. The percentages of Annexin V + 7AAD‐ (early apoptosis) and Annexin V + 7AAD+ (late apoptosis) is indicated. 0.1% DMSO is used as vehicle control. Student’s t‐test was used for the statistical calculations. (E). Heatmap from RNA‐seq analysis displays the scaled expression patterns of genes in PC‐3 cells that were treated with vehicle control, TMX, ISA‐2011B, and combination of TMX and ISA‐2011B. The doses of the agents are indicated. The selected marker genes are listed, red indicating high expression of each marker gene, and blue indicating low expression. (f, g, h and i). Immunoblot analysis on the effect of vehicle control (Ctrl), tamoxifen (TMX), ISA‐2011B (ISA‐2011B) and combination of TMX and ISA‐2011B (Comb.) on expression of ERα, cyclin D1, CDK1, P27, PIP5K1α, pAKT, MMP9 and VEGF in PC‐3 cells. Quantifications of the immunoblots are shown in the right panels. SD ± values indicate means of three independent experiments (for CDK1, control mean = 1.21, 95% CI: 0.96–1.47; *tam*oxifen mean = 0.46, difference = 0.75, 95% CI: 0.27–0.65, *P* = 0.002; ISA‐2011B mean = 0.19, difference = 1.03, 95% CI: 0.15–0.23, *P* < 0.001; combination mean = 0.23, difference = 0.99, 95% CI: 0.07‐0.38, *P* < 0.001). Student’s *t*‐test was used. *P < 0.05 and **P < 0.01 are indicated

To study the inhibitory effect of tamoxifen on castration‐resistant PCa *in vitro* and *in vivo,* and to examine whether it has an on‐target inhibition on ERα‐mediated cancer growth and survival pathways, we treated castration‐resistant PCa cell lines including VCaP and PC‐3 cells, respectively, with tamoxifen at various doses. Tamoxifen at 15, 20, 25, and 30 μm significantly reduced proliferation rate of VCaP cells by 69%, 25%, 17%, and 20% of vehicle‐treated controls, and decreased proliferation rate of PC‐3 cells by 80%, 44%, 19%, and 3% of vehicle‐treated controls (for treatment of VCaP cells with 15 μm tamoxifen, *p* = 0.001, for doses of 20 μm, 25 μm and 30 μm, *P* < 0.001, Fig. 1A; for treatment of PC‐3 cells with 15 μm tamoxifen, *P* = 0.014, for doses of 20 μm, 25 μm and 30 μm, *P* < 0.001, Fig. 1B). We decided to use tamoxifen at 20 μm, this concentration was the IC‐50 dose as determined for the *in vitro* analyses. Since ISA‐2011B is a selective inhibitor of PIP5K1α, a key regulator that acts upstream of PI3K/AKT to promote proliferation and survival of PCa cells [21], we therefore used ISA‐2011B alone or in combination with tamoxifen to assess the effect of tamoxifen on PIP5K1α/PI3K/AKT pathways in PCa cells (Fig. 1C). We observed that tamoxifen treatment led to a dramatic reduction in cell volume and motility of PC‐3 cells as determined by using a live cell holomonitor imaging analysis (Fig. 1C). The tamoxifen effect on reducing the proliferative activity of PC‐3 cells was similar to what was achieved by ISA‐2011B treatment (Fig. 1C), which was reported in our previous studies [21]. Treatment of PC‐3 cells with tamoxifen in combination with ISA‐2011B led to remarkable reduction of cell numbers and altered cell morphology (Fig. 1C). To examine whether tamoxifen treatment may induce apoptosis in PC‐3 cells, we subjected PC‐3 cells that were treated with tamoxifen, ISA‐2011B alone or together to flow cytometry‐based AnnexinV‐7AAD apoptosis assays. Tamoxifen treatment resulted in a significant increase in early apoptosis as represented by the population of Annexin V‐positive and 7AAD‐negative cells (*P* < 0.001, Fig. 1D). The rate of late apoptosis and necrosis as represented by the Annexin V‐positive and 7AAD‐positive populations was also increased in PC‐3 cells after Tamoxifen treatment compared with controls (*P* < 0.001, Fig. 1D). ISA‐2011B at 25 µm also led to significant increase in both early and late apoptosis/necrosis compared with the controls (for both early and late apoptosis, *P* < 0.001, Fig. 1D). Treatment of PC‐3 cells with tamoxifen together with ISA‐2011B led to a significantly higher proportion of early apoptosis and late apoptosis relative of controls (for both early and late apoptosis, *P* < 0.001, Fig. 1D).

To investigate whether tamoxifen has on‐target effect in PCa cells, and to further identify the new targets of tamoxifen, ISA‐2011B, and combination of both agents in PCa cells, we subjected total RNA that were isolated from PC‐3 cells treated with tamoxifen (10 µm), ISA‐2011B (25µ) or combination of both agents to the next‐generation RNA sequencing analysis (RNA‐seq). RNA‐seq analysis is a highly sensitive method. To avoid unspecific changes resulted from the high rate of apoptosis, we used doses of tamoxifen at 10 µm), ISA‐2011B at 25µ in treatment experiments for RNA‐seq analysis. Bioinformatics analysis of the transcriptomic data revealed that *MMP9* was mostly negatively affected by tamoxifen treatment (*P* < 0.001, Fig. 1E), while *PIP5K1A* and *CCND1* were uniquely down‐regulated, and *CDKN1B* was upregulated by ISA‐2011B (*P* < 0.001, Fig. 1E). The key marker genes that are involved in proliferation, survival, and invasion of PCa, including *AKT1, FLT1, FLK1, CDK1, CCNE1,* and *CCNB1,* were down‐regulated by tamoxifen or ISA‐2011B alone (*P* < 0.001, Fig. 1E). Significantly, these key marker genes were down‐regulated by tamoxifen and ISA‐2011B combined together at higher degrees compared with that of tamoxifen or ISA‐2011B alone (*P* < 0.001, Fig. 1E). Taken together, tamoxifen alone or together with ISA‐2011B may exert their effects on PCa cells by specifically targeting a panel of genes that control cancer cell proliferation, survival, and invasion.

To validate our findings from RNA‐seq assays, immunoblot analysis of PC‐3 cells that were treated with tamoxifen, ISA‐2011B alone, or together was performed by using antibodies against a panel of the candidate proteins as mentioned above. Expression of ERα, a specific target of tamoxifen, was firstly examined and was found to be significantly downregulated in tamoxifen‐treated cells compared with controls (control mean = 0.86, 95% CI: 0.84–0.88; tamoxifen mean = 0.63, difference = 0.63, 95% CI: 0.53–0.73, *P* = 0.036, Fig. 1F). Further, expression of cyclin D1, a key target of ERα, was remarkably decreased in tamoxifen‐treated cells compared with controls (control mean = 1.19, 95% CI: 1.18–1.20; tamoxifen mean = 0.01, difference = 1.17, 95% CI: 0.01–0.04, *P* < 0.001; Fig. 1F), suggesting an on‐target engagement of tamoxifen in PC‐3 cells. ISA‐2011B treatment had no pronounced effect on ERα expression, but led to a significantly decrease in cyclin D1 expression compared with controls (control mean = 1.19, ISA‐2011B mean = 0.66, difference = 0.53, 95% CI: 0.47–0.85, *P* = 0.010, Fig. 1F), which was consistent with data from RNA‐seq analysis. Combination treatment using tamoxifen and ISA‐2011B together exhibited more pronounced effect, resulting in diminished cyclin D1 expression in PC‐3 cells (Fig. 1F).

ISA‐2011B treatment resulted in significant reduction in PIP5K1α expression compared with controls (control mean = 0.89, 95% CI: 0.79–0.98; ISA‐2011B mean = 0.69, difference = 0.19, 95% CI: 0.59–0.79, *P* = 0.003), confirming its on‐target engagement on PIP5K1α. Combination treatment using tamoxifen and ISA‐2011B together led to a further decrease in PIP5K1α expression compared with ISA‐2011B alone (*P* = 0.001; Fig. 1G). As expected, expression of phosphorylated pSer‐473 Akt, a downstream target of PIP5K1α, was significantly decreased in PC‐3 cells treated with ISA‐2011B compared with controls (control mean = 1.14, ISA‐2011B mean = 0.57, difference = 0.57, 95% CI: 0.31–0.83, *P* = 0.012). Interestingly, a significant decrease in phosphorylated pSer‐473 Akt was also observed in tamoxifen‐treated cells (control mean = 1.14, 95% CI: 0.86–1.41; tamoxifen mean = 0.53, difference = 0.61, 95% CI: 0.13–0.92, *P* = 0.045) and was further decreased in cells treated with tamoxifen and ISA‐2011B together (*P* < 0.001; Fig. 1G). Further, expression of CDK1 and P27, the downstream effectors of AKT was significantly affected in PC‐3 cells treated with tamoxifen, ISA‐2011B alone or in combination compared with controls (for CDK1, tamoxifen, *P* = 0.002; for ISA‐2011B, *P* < 0.001; for combination treatment *P* < 0.001; Fig. 1H). Taken together, the data obtained from immunoblot analysis is largely in agreement with what was observed using RNA‐seq analysis, suggesting that tamoxifen may directly target AKT pathway, and that tamoxifen and ISA‐2011B may have synergistic effect on a panel of targets including cyclin D1, AKT pathways.

Consistent with previous reported studies, ISA‐2011B treatment led to significant decrease in expression of both MMP9 and VEGF (for MMP9, *P* = 0.014; for VEGF, *P* = 0.026; Fig. 1I). Combination of tamoxifen and ISA‐2011B had significantly inhibitory effects on MMP9 expression (*P* < 0.025, Fig. 1I), and on expression of VEGF (*P* < 0.031, Fig. 1I). Taken together, the data from immunoblot analysis validated majority of our findings from RNA‐seq data, and further suggest that tamoxifen and ISA‐2011B may have synergistic inhibitory effect on proliferation, survival, and invasive pathways of PCa cells.

### 3.2 Tamoxifen alone or in combination with PIP5K1α inhibitor ISA‐2011B suppresses tumor growth in xenograft mouse models

**Fig. 2:**
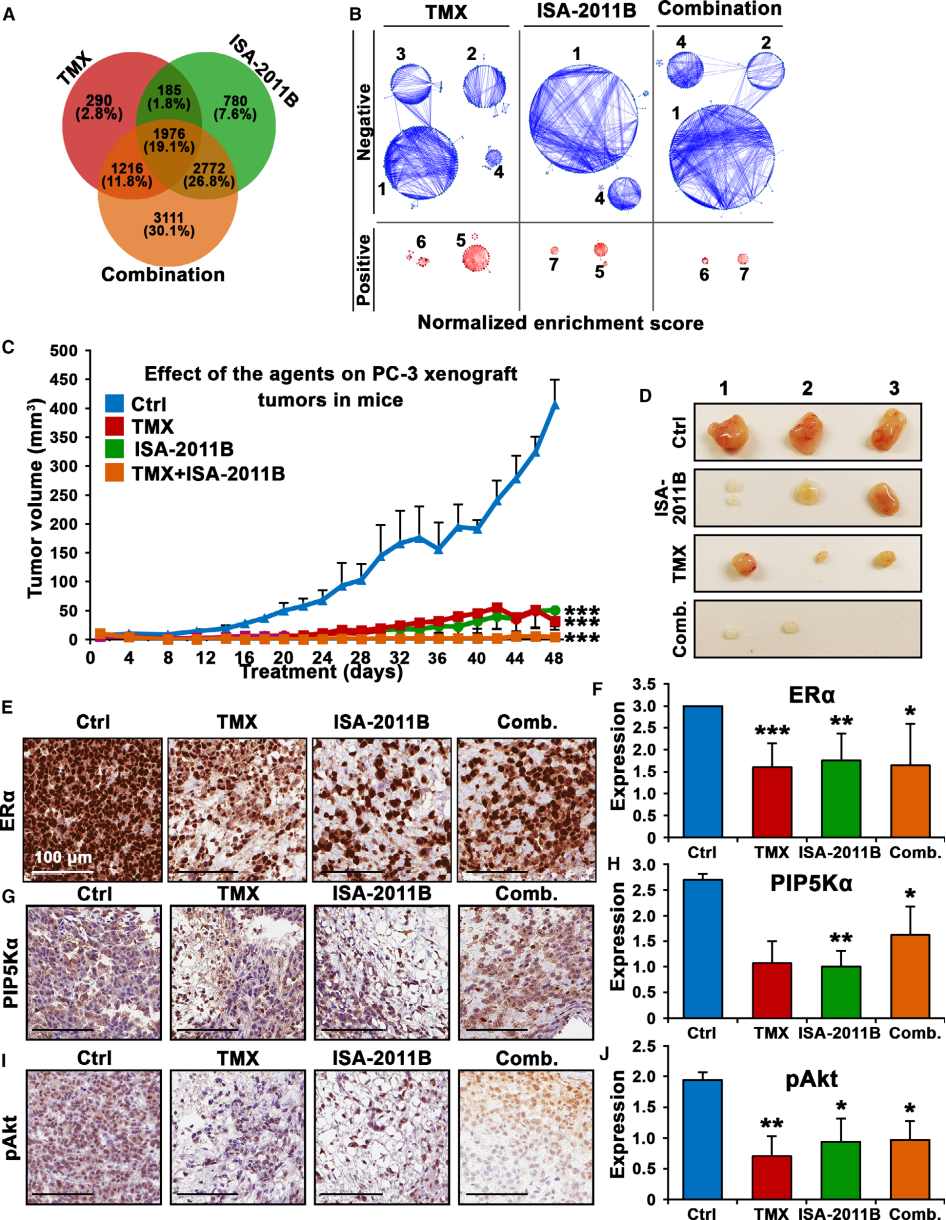
The inhibitory effects of tamoxifen, ISA‐2011B and combination of tamoxifen and ISA‐2011B on downstream targets in PCa cell lines and on growth of PCa xenograft tumors in mice. (A). The pie chart shows the number of genes in each cluster of the network of genes that have been affected by tamoxifen (TMX), ISA‐2011B and combination of TMX and ISA‐2011B. (B). The gene sets enrichment analyses data are show. The network of genes (25% in each PC3 cells that have been affected tamoxifen (TMX), ISA‐2011B and combination of TMX and ISA‐2011B. The numbers indicate the biological roles of each gene network. 1: Cell cycle; 2: Protein synthesis; 3: DNA synthesis; 4: ATP synthesis; 5: Metabolism; 6: Cell death; 7: ER stress response. The significantly negatively affected/down‐regulated networks are in blue color. The significantly positively affected/ upregulated network is in red color. (C) Growth of tumor xenografts treated with vehicle (Ctrl), TMX, ISA‐2011B and combination of ISA‐2011B and TMX. Treatment started on day 1 for every second day and ended on day 48. Mean tumor volumes and upper 95% confidence intervals are shown. Both ANOVA test and Student’s t‐test is used. ***P < 0.001. (D) Representative images of tumors from each group are shown. (E–J). Representative microphotographs of immunohistochemical analysis of the marker protein expression in tumors from each treatment group. Vehicle control (Ctrl), tamoxifen (TMX), ISA‐2011B (ISA‐2011B), and combination treatment of tamoxifen and ISA‐2011B (com.) are indicated. Expression of ERα, PIP5K1α, phosphorylated AKT in tumors from each treatment group was quantified. The scale bars showing 100 µm are indicated. Quantifications of the staining intensities of various biomarkers are shown in the right panels. Student’s *t*‐test was used. *P < 0.05 and **P < 0.01 are indicated.

Consistent with the data mentioned above, we found that a final list of 3667 candidate genes that were affected by tamoxifen, and 5712 candidate genes affected by ISA‐2011B, and 9074 genes affected by combination of tamoxifen and ISA‐2011B ( under PDR cutoff, *P* < 0.05 Fig. 2A, Table S1). Gene ontology enrichment analysis of biological process further confirmed a robust relationship between candidate gene network and the effects of tamoxifen, ISA‐2011B, and combination of both agents (Fig. 2A). We found that the common gene networks that are responsible for cell cycle, ATP synthesis, metabolisms were significant affected by tamoxifen and ISA‐2011B. Interestingly, gene networks that responsible for apoptosis, DNA and protein synthesis appeared to be preferentially affected by tamoxifen, while endoplasmic reticulum (ER) stress network was preferentially affected by ISA‐2011B (Fig. 2B and Table S1). Combination of tamoxifen and ISA‐2011B showed synergistic effect on cell cycle, protein synthesis, ATP synthesis, ER stress, and cell death (Fig. 2B). Thus, we identified the unique and common gene network that were targeted by tamoxifen, ISA‐2011B and combination of both agents in PCa cells.

To further test the biological and clinical relevance of tamoxifen, combination of tamoxifen and ISA‐2011B together for targeted treatment of castration‐resistant PCa, we employed xenograft mouse model bearing castration‐resistant tumor PC‐3 with elevated level of ERα and constitutively high levels of PIP5K1α/pSer‐473 Akt. The mice were randomized into four groups and treated accordingly with vehicle control, tamoxifen (40 mg/kg), ISA‐2011B (40 mg/kg), or tamoxifen and ISA‐2011B at half dose of each via intraperitoneal administration, every second day for a total of 48 days (Fig. 2C). At the end of treatment, the mean volumes of tumors treated with tamoxifen, ISA‐2011B or combination of tamoxifen and ISA‐2011B respectively were only 7.9%, 12.5%, and 1% of the mean volume of tumors that were treated with vehicle control (mean volume for vehicle‐treated control = 406.8 mm^3^, 95% CI: 323.3–490.3; mean volume for tamoxifen‐treated = 32.2 mm^3^, difference = 377.0 mm^3^; 95% CI: 2.4–62.0 mm^3^, *P* < 0.001, *n* = 5; mean volume for ISA‐2011B‐treated = 51.0 mm^3^, difference = 351.5 mm^3^; 95% CI: −4.2–106.3 mm^3^, *P* < 0.001, *n* = 5; mean volume for combination‐treated = 4.1 mm^3^, difference = 398.8 mm^3^; 95% CI; 3.9–12.1 mm^3^, *P* < 0.001, *n* = 4; Fig. 2C). Tumors from two mice out of the combination treatment group disappeared (Fig. 2C, D), suggesting that tamoxifen in combination with ISA‐2011B had pronounced inhibitory effect on tumor growth as compared with that of single agent. Mice were well tolerated to tamoxifen treatment alone or in combination with ISA‐2011B.

Immunohistochemical analysis was further performed to examine the effects of tamoxifen, ISA‐2011B alone or in combination on the targeted proteins including ERα, PIP5K1α, pSer‐473 Akt, VEGF, and VEGFR2 in tumors from each treatment group. ERα expression was significantly decreased in tumors treated with tamoxifen, ISA‐2011B or combination of tamoxifen and ISA‐2011B, compared with that of controls (*P* < 0.001, Fig. 2E,F). As expected, PIP5K1α expression was significantly decreased in tumors treated with ISA‐2011B (*P* = 0.016, Fig. 2G,H). Expression of pSer‐473 Akt was markedly reduced in tumors treated with tamoxifen, ISA‐2011B or combination of tamoxifen and ISA‐2011B as compared with that of controls (for tamoxifen‐treated *P* = 0.003; for ISA‐2011B‐treated *P* = 0.020; and combination treated, *P* = 0.03 Fig. 2I,J). Similar to ISA‐2011B, tamoxifen treatment reduced expression of VEGF and its receptor VEGFR2 in xenograft tumors, so as the combination treatment (for VEGF, all treatment showed *P* < 0.001, and for VEGFR2, all treatment showed *P* < 0.001; Fig S1). Taken together, tamoxifen exerts its effect to suppress growth of PCa tumor *in vivo*, probably by inhibiting ERα and AKT pathways. Combined treatment using tamoxifen together with ISA‐2011B led to regression of tumor growth in mice, probably by synergistically blocking elevated ERα and PIP5K1α/AKT pathways. Taken together, our *in vivo* data is in agreement with what was obtained from cell line‐based studies mentioned above.

#### 3.2.1 ESR1 mRNA expression in distinct subgroup of prostate cancer patients and its association with patient outcome

**Fig. 3:**
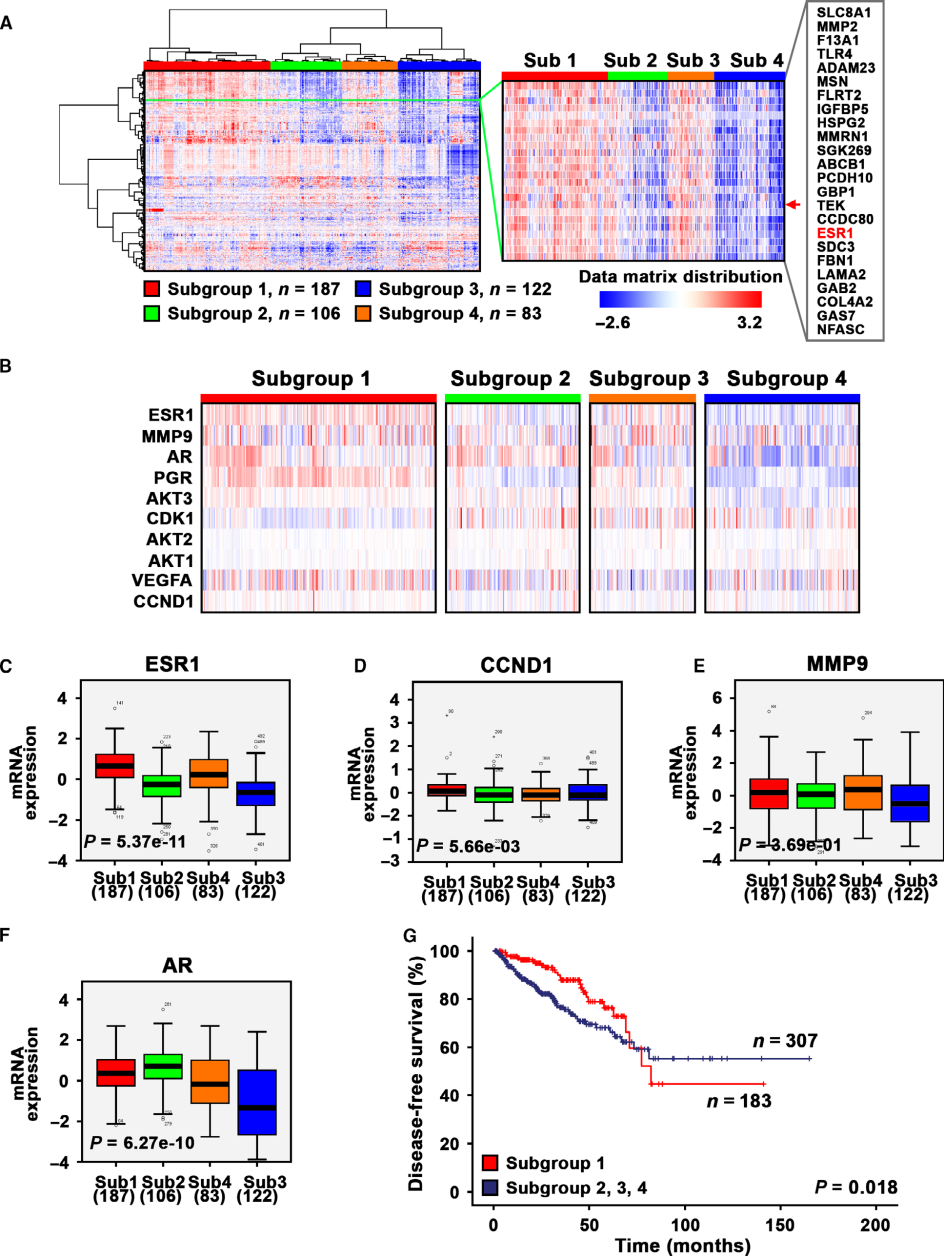
Clinical importance and gene expression signatures of estrogen‐associated signaling pathways in primary cancer tissues from PCa patients. (A) Hierarchical clustering of primary cancer tissues from a cohort of 498 PCa patients was performed based on their multilayer expression patterns of marker gene signatures. A dendrogram of the tumors from patients is shown at the top and left, with four distinct subclusters/subgroups denoted. The gene names are listed on the right. Red indicating high expression of each gene, and blue indicating low expression. (B) The magnified heatmap showing expression patterns of the key marker genes including ESR1, *MMP9, AR*, progesterone receptor (*PRG*), *AKT3, CDK1, AKT2, AKT1*, *VEGFA* and *CCND1*, which are the key marker genes that control proliferation, survival and invasion signaling pathways. (C–F). Box plots show that *ESR1*, *CCND1*, *MMP9* and *AR* are significantly differentially expressed among the four subclusters/subgroups. The statistical program ANOVA was used. *P* values indicate significant differences among the four subclusters/subgroups are shown. (G) Kaplan–Meier survival curve revealed that patients from subcluster/subgroup 1 (*n* = 183) suffered poorer disease‐free survival (DFS) as compared to those from subclusters/subgroups 2–4 (*n* = 307). Differences in disease‐free survivals between two groups were calculated by using SPSS log‐rank statistical analysis. *P* value is indicated, *P* = 0.018.

As reported in our previous studies, we found that clinically relevant gene profiles that are associated with PIP5K1α/AKT in subgroups of patients, and that elevated level of PIP5K1α was associated with poorer patient outcome [16] We therefore examined expression profiles of the target genes of tamoxifen identified by RNA‐seq as mentioned above by using primary tumors from PCa patients. To this end, we extracted hierarchical clustering data analyzed by iCluster analysis using primary cancer tissues from 498 PCa patients from The Cancer Genomic Atlas (TCGA), publicly available in cBioPortal databases [33, 34, 35]. The gene signatures allowed to segregate the primary tumors into four molecularly distinct subclusters (Fig. 3A). Further, *ESR1, MMP9, AR,* progesterone receptor (*PGR*), *AKT3, CDK1, AKT2, AKT1, VEGFA,* and *CCND1* were among the genes that exhibited differential expression signatures that divided primary tumors into four distinguished molecular subtypes (Fig. 3A,B, Fig. S2). Statistical analysis further revealed that genes that were targeted by tamoxifen or ISA‐2011B including *ESR1*, *CCND1*, *MMP9*, *AR* showed differential expression in subgroup 1, which was significantly different compared with remaining subgroups (for these mentioned genes, all *P values are* < 0.001; Fig. 3C–F). This suggests that clinically relevant gene profiles that are targeted by tamoxifen and ISA‐2011B are expressed uniquely by subgroups of patients. To examine whether the subgroup 1 with differential expression pattern of *ESR1*, *CCND1*, *MMP9*, *AR* and other key marker genes might be associated with worse clinical outcome compared with subgroups 2, 3 and 4, we performed Kaplan–Meier survival analysis. We found that patients who had primary tumors belonging to subgroup 1 (*n* = 183) suffered significantly poorer disease‐free survivals (DFS) as compared to those from subgroups 2, 3 and 4 (*n* = 307), and this difference was statistically significant (*P* = 0.018) (Fig. 3G). These results indicate that clinically relevant subgroups of PCa patients may have unique gene signatures within the targeted frames of tamoxifen, ISA‐2011B or combination of both agents.

### 3.3 ERα protein expression and its correlation with PIP5K1 in primary and metastatic PCa from patient cohorts

**Fig. 4:**
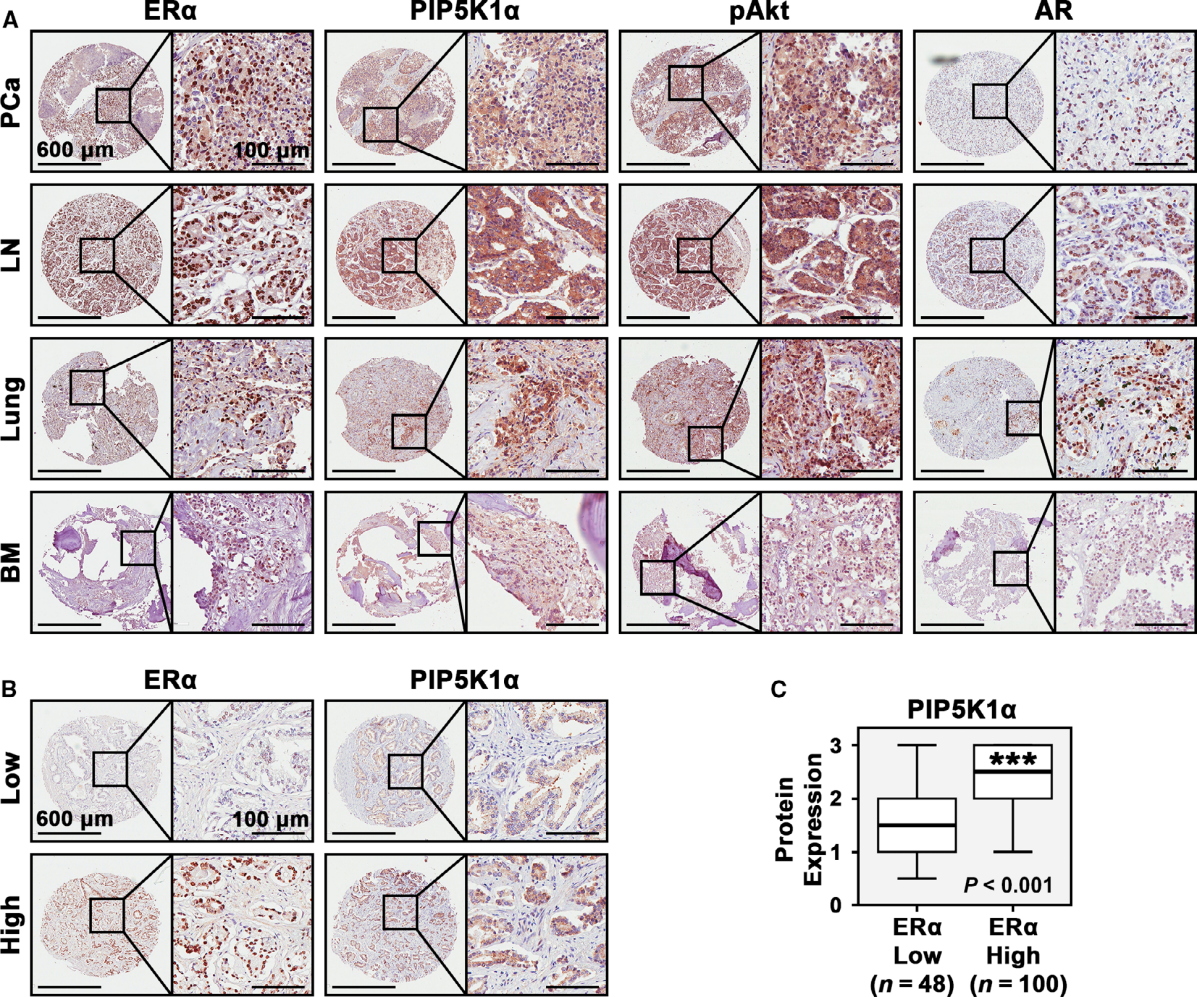
Evaluation of the expression of PIP5K1α associated pathways and estrogen signaling in primary cancer tissues and metastatic lesions from patients with PCa and distant metastasis. (a). Immunohistochemical analysis of ERα expression along with expression of PIP5K1α, Phosphorylated Akt and AR in primary PCa tissues (*n* = 113), and in metastatic lesions in lymph nodes (LN) and lungs (Lung) bones/bone marrows (BM) (*n* = 43). Representative microphotographs of PCa tissues stained with antibodies against the proteins as mentioned above are shown. Scale bars of 600 μm and 100 μm are indicated. (b) Representative microphotographs revealed expression levels and subcellular localization of ERα and PIP5K1α in primary cancer tissues of low vs. high Gleason grades. Scale bars of 600 μm and 100 μm are indicated. (c). Box plots showed primary PCa that expressed high ERα also had high level of PIP5K1α expression (*P* < 0.001). Student’s *t*‐test was used for the statistical significance in calculations.

We next examined protein expression of the candidate genes as mentioned above by using primary and metastatic lesions from our own patient cohorts. Immunohistochemical analysis revealed that ERα protein was highly expressed in primary tumor tissues and in metastatic lesions from lymph nodes, lung, and bone marrow of PCa patients (Fig. 4A). PIP5K1α, pAKT and AR was also highly expressed in primary tumor tissues and metastatic lesions from PCa patients in these cohorts. Interestingly, we found a statistically significant correlation between ERα and PIP5K1α expression in primary cancer tissues and metastatic lesions (*r* ^2^ = 0.513, *P* < 0.001, Fig. 4B,C). The correlation between the expression of ERα and PIP5K1α in tumor tissues was also illustrated using the correlation scatter plot (Fig. S4). Given that PIP5K1α is a key factor that contributes to progression of PCa, this finding suggests that ERα may be functionally involved in PCa progression and serves as a target for tamoxifen treatment of PCa.

### 3.4 The effect of ERα overexpression on PCa cancer cell lines

**Fig. 5:**
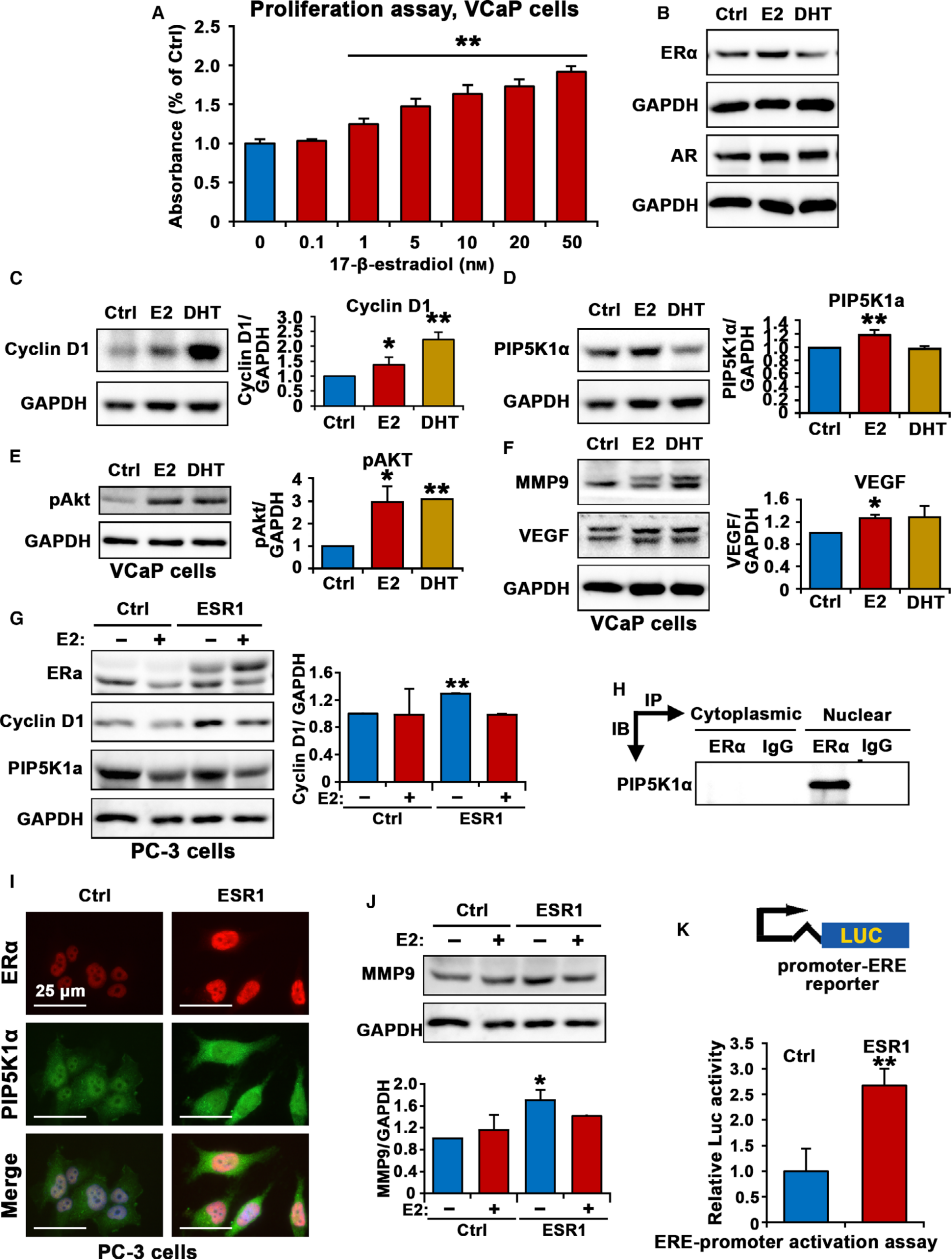
The role of estrogen and ERα signaling and its association with PIP5K1α/PI3K/AKT signaling pathways in VCaP cells and PC‐3 cells. (A). Dose‐dependent effects of *17‐β estradiol* on the proliferation of VaCp cells are shown. *17‐β estradiol* at 0.1 up to 50 nM was used. (B and C). Immunoblot analysis show the effect of *17‐β estradiol* (E2) or DHT on expression of ERα, AR and cyclin D1 in VCaP cells. Quantifications of the immunoblots for cyclin D1 expression are shown in the right panel. (for cyclin D1, control mean = 0.25, CI 0.22‐0.27; *17β*‐*Estradiol*‐treated mean = 0.34, difference = 0.10, 95% CI 0.31–0.37, *P* = 0.019; DHT‐treated mean = 0.52, difference = 0.27, 95% CI = 0.49‐0.54, *p* < 0.001). SD ± values indicate means of three independent experiments. *P < 0.05 and **P < 0.01 are indicated. (D and E). Immunoblot analysis show the effect of *17‐β estradiol* (E2) or DHT on expression of PIP5K1α and pAKT. Quantifications of the immunoblots for PIP5K1α and pAKT expression are shown in the right panels (for PIP5K1α, control mean = 0.92, 95% CI 0.88–0.96; *17β*‐*Estradiol*‐treated mean = 1.06, difference = 0.14, 95% CI 1.05–1.08, *p* = 0.002). (F). Immunoblot analysis show the effect of *17‐β estradiol* (E2) or DHT on expression of MMP9 and VEGF in VCaP cells. For VEGF, control mean = 0.44, 95% CI: 0.41–0.47; *17β*‐*Estradiol*‐treated mean = 0.56, difference = 0.12, 95%CI: 0.55–0.57. *P < 0.05 and **P < 0.01 are indicated. (G). Immunoblot analysis show the effect of *17‐β estradiol* (E2) (+) or vehicle control (−) on ERα, cyclin D1 and PIP5K1α in PC‐3 cells that were transfected with empty control vector (Ctrl) or vector containing ESR1 (ESR1). (H) The lysates from the cytoplasmic and nuclear compartments of PC‐3 were subjected to immunoprecipitation (IP) assay. Antibody against ERα was used to pull down and antibody to IgG was used as a negative control. Antibody against PIP5K1α was used for immunoblot analysis (IB). (I) Representative immunofluorescent microphotographs showed the subcellular localization of ERα in red and PIP5K1α in PC‐3 cells transfected with ESR1 (ESR1) or control vector (Ctrl). The scale bar of 25 µm is indicated. (J) Immunoblot analysis shows the effect of E2 treatment on MMP9 expression in PC‐3 cells transfected with ESR1 or control vector. Quantifications of the immunoblots for MMP9 expression are shown in the panel below. *P < 0.05 is indicated. (K) Effect of induced ESR1 or control (Ctrl) on the activity of estrogen responsive genes containing ERE motifs as assessed using luciferase assay. SD ± values indicate means of three independent experiments. *P < 0.05 is indicated. Student’s t‐test was used in the analysis shown in this figure.

We found that ESR1 mRNA was expressed in PC‐3 cells by using targeted sequencing analysis (Fig. S3). Next, we investigated whether elevated expression of ERα might be associated with increased growth and survivals of PCa cells. We subjected VCaP cells to *17β*‐*Estradiol* treatment at increasing doses ranging from 0.1 nm up to 50 nm for 48 h. The proliferation rate was increased by 63% in VCaP cells treated with *17β*‐*Estradiol* at 10 nm relative of controls, and the effect of *17β*‐*Estradiol* on VCaP cell proliferation was dose‐dependent (*P* = 0.001, Fig. 5A). Consistent with its positive effect on proliferation, *17β*‐*Estradiol* treatment significantly increased cyclin D1 expression by 39% relative of controls (*P* = 0.019, Fig. 5B,C). This was similar to the effect of DHT, where cyclin D1 expression increased by 123% after DHT treatment, compared with that of controls (*P* < 0.001, Fig. 5C). Constitutive activation of PIP5K1α/PI3K/Akt pathways are involved in progression and poor response to androgen deprivation therapy in PCa [21, 36], we therefore examined effect of *17β*‐*Estradiol* treatment on expression of PIP5K1α and phosphorylated Ser‐473 AKT. *17β*‐*Estradiol* treatment led to a significant increase in PIP5K1α and an increase in phosphorylated Ser‐473 AKT, by 194% increase relative of the controls (for PIP5K1α, *P* = 0.002; for Ser‐473 Akt, control mean expression value = 0.12, 95% CI: 0.07–0.17; *17β*‐*Estradiol*‐treated mean value = 0.33, difference = 0.21, 95% CI 0.30–0.36, *P* = 0.032, Fig. 5D and e). *17β*‐*Estradiol* also markedly increased MMP9 and VEGF expression compared with controls (for VEGF, *P* = 0.019, Fig. 5F). These data show that *17β*‐*Estradiol* treatment is able to increase proliferative ability of PC‐3 cells, and leads to upregulation of ERα and its associated cyclin D1, PIP5K1α, phosphorylated AKT, and MMP9/VEGF in PCa cells.

To further study a functional role of ERα as a transcriptional factor in mediating downstream target genes involved in growth, survival and invasion in PCa cells, we induced overexpression of ERα in PC‐3 cells that lack AR expression, and treated the cells with *17β*‐*Estradiol* to further stimulate the activation of ERα. ERα overexpression alone was sufficient to upregulate cyclin D1 expression in PC‐3 cells compared with controls (*P* < 0.001, Fig. 5G). ERα overexpression also enhanced expression of PIP5K1α in PC‐3 cells (Fig. 5G). However, *17β*‐*Estradiol* treatment had no significant effect on expression of cyclin D1 and PIP5K1α (Fig. 5G). Since PC‐3 cells were cultured in phenol‐red free medium containing 10% charcoal‐stripped serum, in which the steroid hormones, eg, estrogens and androgens were depleted, thus this finding suggests that ERα and its associated pathways may be independent of *17β*‐*Estradiol* in PC‐3 cells that lack functional AR. Interestingly, immunoprecipitation assays revealed that ERα formed protein–protein complexes with PIP5K1α exclusively in the nuclear compartment of PC‐3 cells (Fig. 5H). Immunofluorescence analysis was performed to assess the subcellular localization of ERα and PIP5K1α expression in PC‐3 cells that were transfected with ERα or control vectors. We found that ERα was predominantly localized in the nucleus, and ERα expression. PIP5K1α expression was observed in both nucleus and cytoplasmic compartments in PC‐3 cells and PIP5K1α expression was markedly enhanced in ERα‐transfected cells as compared with that of controls (Fig. 5I). ERα overexpression also led to a significant increase in MMP9 expression, a key marker of invasiveness by 70% relative of controls (control mean expression value = 0.26, 95% CI: 0.11.0–42; mean value in ERα overexpressing cells = 0.45, difference = 0.18, 95% CI: 0.42–0.48, *P* = 0.036, Fig. 5J).

To elucidate the functional consequences of ERα overexpression in PCa cells, we carried out dual‐luciferase (Luc) assays by using control and (ERE) reporter plasmids. ERα overexpression induced ERE reporter luciferase activity by 167.1% as compared with that of controls in PC‐3 cells (*P* = 0.006, Fig. 5K). Thus, ERα is able to mediate the activity of the key factors that contribute to proliferation, survival and invasion in castration‐resistant PCa cells.

### 3.5 Depletion of tumor‐associated myeloid cells sensitizes tamoxifen effect

**Fig. 6:**
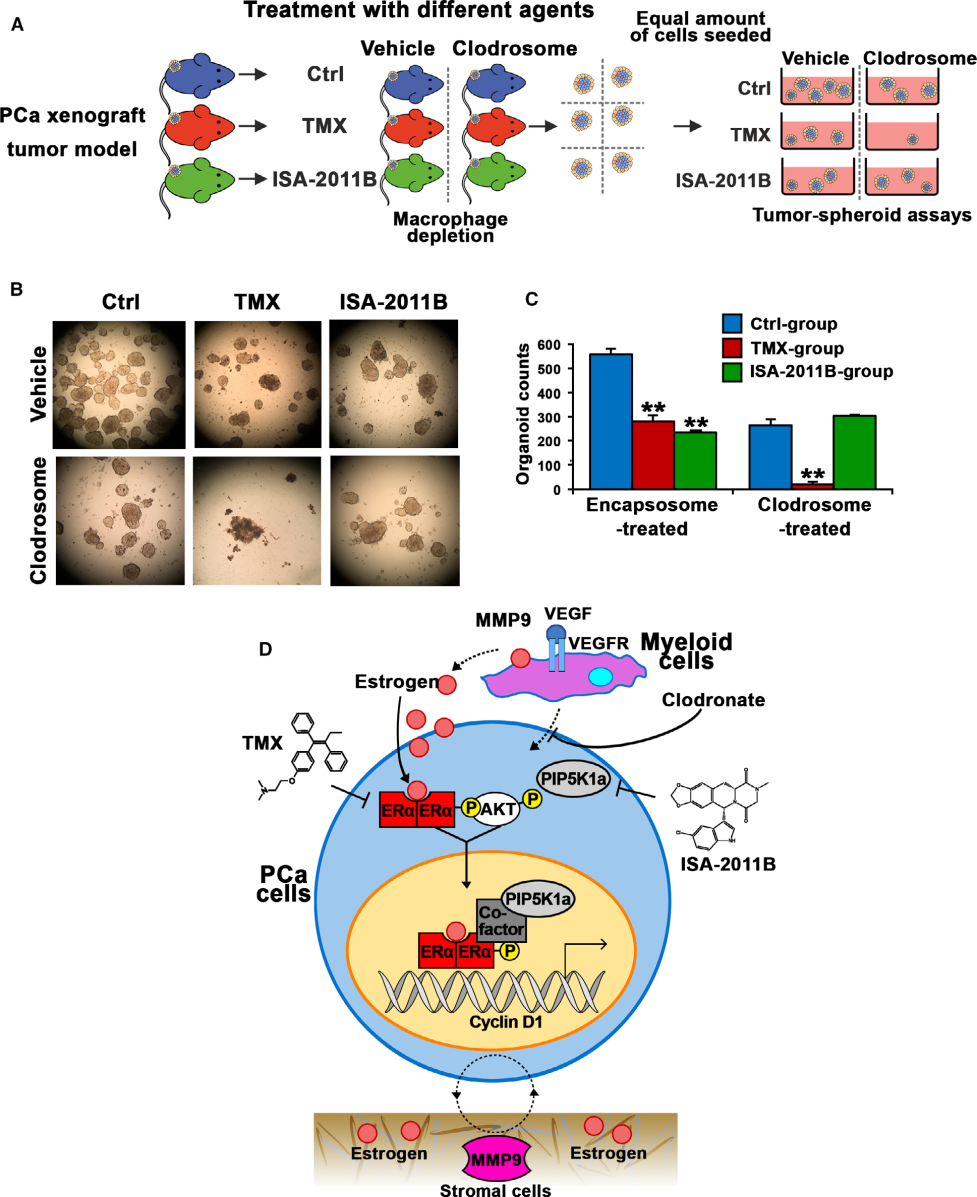
The effect of depletion of tumor‐associated macrophages on tamoxifen treatment of aggressive PCa in xenograft mouse models and tumor spheroid models, and the cellular mechanisms that mediate on‐target effect of TMX, ISA‐2011B and combinations of TMX and ISA‐2011B in PCa cells. (A) A schematic model illustrate the experimental procedure. Xenograft mice bearing PC‐3 tumors of approximately 400 mm^3^ volume were randomized and were grouped into treatment groups that were treated either with vehicle control, TMX, or ISA‐2011B. The treatment groups were then divided into two subgroups that were either treated with encapsosome as vehicle control (Vehicle) or with Clodronate (clodrosome). At end of the experiments, tumors were removed. Single cell were isolated from the tumors and were subjected to tumor spheroid analysis. (B). Representative images were taken under phase contrast microscope (Olympus) with the magnifications 10x20 objective lens. The images of tumor spheroids derived from primary tumors from mice of each treatment groups (3–4 mice per group) are illustrated. (C) The counts of tumor spheroids from each group are shown. SD ± values indicate means of three independent experiments. Student’s *t*‐test was used in the analysis. *P < 0.05 and **P < 0.01 are indicated. (D) A schematic model illustrating the role of estrogen and its receptor ERα in regulation of transcriptional activities of its target genes such as cyclin D1. ERα may act on PIP5K1α/Akt through protein‐protein interaction with PIP5K1α in the nucleus. Tamoxifen, ISA‐2011B have on‐target effects on their common downstream targets. The combination treatment using tamoxifen and ISA‐2011B, or combination of tamoxifen with clodronate may have synergistic inhibitory effect on PCa cells.

It is known that estrogen is converted from androgen by aromatase. Depletion of androgen by androgen deprivation therapy, also leads to depletion of estrogen in PCa patients [37]. However, we previously found that bone marrow‐derived myeloid cells produce high level of estrogen are capable of supporting growth of PC‐3M cells, thus suggesting that ERα in PCa cells may utilize its ligands from bone marrow‐derived cells [11]. To this end, we hypothesized that depletion of tumor‐associated bone marrow‐derived cells in particular macrophages by Clodronate may sensitize inhibitory effect of tamoxifen. Clodronate is a bisphosphonate that has been shown to improve survival of breast cancer patients [38]. Intriguingly, clodronate was shown to improve overall survival in men with metastatic PCa who were starting hormone therapy [39]. To test our hypothesis, we established aggressive PC‐3 xenograft tumors models by allowing subcutaneously implanted tumors to grow into large size with the mean volume of approximately 400 mm^3^ in mice. We then used clodronate to selectively deplete tumor‐associated macrophages via intratumor injection in combination with intraperitoneal administration of tamoxifen or ISA‐2011B in tumor‐bearing mice. After two weeks of treatment, the tumors were removed and were made into single cell suspensions. Equal amounts of cells from each treatment group were subjected to tumor spheroid formation assays in 3D models (Fig. 6A). We found that there were significantly less tumor spheroids that were formed from cells extracted from tumors of mice treated with tamoxifen as compared with vehicle controls (*P* = 0.001, Fig. 6B,C). Similarly, there were also significantly less numbers of tumor spheroids from tumors of ISA‐2011B‐treated mice group compared with that of controls (*P* < 0.001, Fig. 6B,C). Interestingly, clodronate treatment alone reduced the ability of tumor cells to form tumor spheroids compared with that of controls (*P* < 0.001, Fig. 6B,C). Strikingly, there were barely tumor spheroids formation from tumors that were treated with both tamoxifen and clodronate, and this was statistically significant compared with controls (*P* = 0.003, Fig. 6B,C). In contrast, the numbers of tumor spheroids remained similar between ISA‐2011B‐treated alone compared with clodronate and ISA‐2011B in combination (Fig. 6B,C). These data suggest that tamoxifen may be used together with clodronate to effectively inhibit growth of PCa (Fig. 6D).

## 4 Discussion

The castrate‐resistant PCa represents an incurable and ultimately fatal stage, with a few treatment options. In this study, we applied novel therapeutic approaches to suppress growth of PCa by using tamoxifen alone or in combination with PIP5K1α inhibitor ISA‐2011B to targeting estrogen/ERα and PIP5K1α/Akt pathways in cell line and in mouse models. Pathways analysis of tamoxifen treatment effects have been performed using breast cancer cell lines, and cell cycle and cholesterol biosynthesis were the targets of tamoxifen [40]. Here, we uncovered previously unrealized diversity in gene expression relating to treatment effects of tamoxifen, ISA‐2011B or combination of tamoxifen and ISA‐2011B on PCa cells. By analyzing gene signatures in PCa cells that have been treated with tamoxifen, ISA‐2011B and the combination of both agents, we suggested a potential new approach to treat advanced CRPC.

In the present study, we analyzed multilayer expression signatures of marker genes in primary cancer tissues from 498 PCa patients by using iCluster analysis. We identified four distinguish molecular subtypes of primary tumors based on differential expression signatures such as ERα, AR, and PI3K/Akt signaling pathways. Our findings suggest that many of patients who had elevated expression of tumor‐specific signatures such as ESR1, AR, VEGF, AKT1/2/3, CCND1, CDK1, and MMP9 had significantly poorer disease‐free survivals compared with the remaining subgroups.

Estrogen production is increased throughout the body during androgen deprivation therapy (ADT). Since ADT, nonsteroidal anti‐androgens will block AR, which in turn through a feedback loop increase the secretion of luteinizing hormone (LH), leading to increased estrogen production, and elevated expression of ERα. Thus, as a direct side‐effect, patients suffer gynecomastia or/and breast pain due to ERα expression in breast tissues. Enzalutamide, an androgen receptor signaling inhibitor was shown to be associated with a 49% rate of gynecomastia within 2 years. Tamoxifen has been used as drug intervention to block the ERα in breast tissues and the data from 8 clinical randomized trials revealed that tamoxifen is well tolerated by PCa patients[41].

However, given the facts that ERα overexpression can occur due to the disturbed balance between estrogens and androgens, it remained unexplored regarding to ERα expression, its role and underlying mechanisms in PCa progression. As illustrated by our proposed model in Fig. 6D, we have identified tumor‐intrinsic ERα as tumor‐promoting factor for PCa growth. Our model suggests that ERα signaling promotes growth by increasing downstream cyclin D1 and pAKT, PIP5K1α, and the effectors of pAKT including CDK1 and P27. We show that ERα and PIP5K1α formed protein complexes in the nuclear compartment, and affected PIP5K1α and phosphorylated AKT in PCa cells. These findings suggest that ERα acts as a transcriptional factor on its target genes such as cyclin D1, but ERα may also be able to mediate function of the proteins such as PIP5K1α and AKT at post‐translational level in PCa cells. In the *in vivo* xenograft mouse models, we used PC‐3 cells model, as these cells lack AR, this allowed us to specifically study the ERα‐mediated effect in PCa cells. Our findings further revealed the mechanisms suggesting that ERα is a key target that mediates therapeutic response of tamoxifen in PCa. In this study, we observed that PC‐3 cells that lack functional androgen receptor (AR) responded to *17β‐Estradiol* in different ways as compared to that of VCaP cells that express functional AR. Our findings suggest that AR may play an important role in mediating the effects of *17β‐Estradiol* in PCa cells. It will be interesting to apply VCaP cells to conduct *in vivo* studies to gain deeper understanding of the underlying mechanisms in the near future.

In the present study, we unrevealed the effect of tamoxifen on panel of genes and network that are associated with ERα and PIP5K1α/AKT that are responsible PCa growth and invasion. In the present study, treatment of xenograft mice bearing PCa tumors with tamoxifen combined with ISA‐2011B resulted in regression of tumor growth. A synergistic effect of tamoxifen and ISA‐2011B on induction of apoptosis and inhibition of tumor growth was observed. Combination treatment greatly inhibited ERα, PIP5K1α, pAKT, VEGF and VEGFR2 expression in tumors, which reversely correlated with the elevated levels of these key factors in aggressive tumors. The deeper understanding of the effect of therapeutic response to tamoxifen, ISA‐2011B and combination of both agents may suggest a new approach in PCa therapy. Our data herein provide the rationale for development of these agents for castration‐resistant PCa.

Data from the co‐culture experiments in our reported studies have provided evidence suggesting that ERα expression in PCa can be stimulated by estrogen from the bone marrow cells. Thus, bone marrow cells promote growth of ERα‐positive PCa cells by providing estrogen [11]. In this study, we depleted tumor‐associated macrophages by using intratumor administration of clodronate given to xenograft mice‐bearing aggressive and large tumors, as it is well known that macrophages produce high level of estrogen. We found that depletion of tumor‐associated macrophages sensitized the effect of tamoxifen and sufficiently blocked ability of PCa cells to form tumor spheroids. Treatment of tumor‐bearing xenograft mice with tamoxifen and clodronate in combination showed greater inhibitory effect as compared to the mono‐treatment. Our results highlighted an important role of tumor‐associated macrophages/tumor microenvironment in PCa growth. This finding also suggests that tamoxifen may be used together with clodronate to effectively inhibit growth and progression of PCa. It will be interesting to systematically identify the target genes of tamoxifen and clodronate by performing RNA‐seq in using both cell line systems and tumors in a side‐by‐side manner in the near future.

## 5 Conclusions

In relation to personalized medicine, we integrate preclinical and functional studies with the metagenomics and bioinformatics from clinical studies to better predict the drug and targets‐mediated effects. Our study suggests that tamoxifen alone or in combination with ISA‐2011B may provide new therapeutic opportunities, and the targets that we discovered and validated can be used as biomarkers to identify patients at risk of metastasis and aid in early stratification of candidates.

## Conflict interest

The authors declare no conflict of interest.

## Author contributions

JS, TW, and JLP designed experiments. JS, TW, and ASSK performed experiments. JS, TW, AKMFM, AS, and ASSK performed data analysis. JS, TW, and JLP contributed to manuscript writing. All authors contributed to writing, editing, and final approval of the manuscript.

### Peer Review

The peer review history for this article is available at https://publons.com/publon/10.1002/1878‐0261.12873.

## Supporting information


**Fig S1.** The effect of tamoxifen and ISA‐2011B alone or in combination on VEGF and VEGFR2 expression in PC‐3 xenograft tumors in mice.

**Fig S2.** Clinical importance and gene expression signatures of estrogen‐associated signaling pathways in primary cancer tissues from PCa patients.

**Fig S3.** The presence of ESR1 mRNA in PC‐3 cells.

**Fig S4.** The correlation scatter plot.

**Table S1.** Lists of functiona pathways that are significantly affected by tamoxifen‐treatment alone, ISA‐2011B‐treatment alone, or combination treatment using tamoxifen together with ISA‐2011B in PC‐3 cells.

## Data Availability

All data analyzed for this study are included in this published article and its supplemental information files. The raw data can be provided upon request to corresponding authors.
