# Supplementary figures and images for: Targeted inhibition of ERα signaling and PIP5K1α/Akt pathways in castration‐resistant prostate cancer
Source: Mol Oncol. 2020 Dec 16;15(4):968–86. doi: 10.1002/1878-0261.12873 (PMC8024724; doi:10.1002/1878-0261.12873)

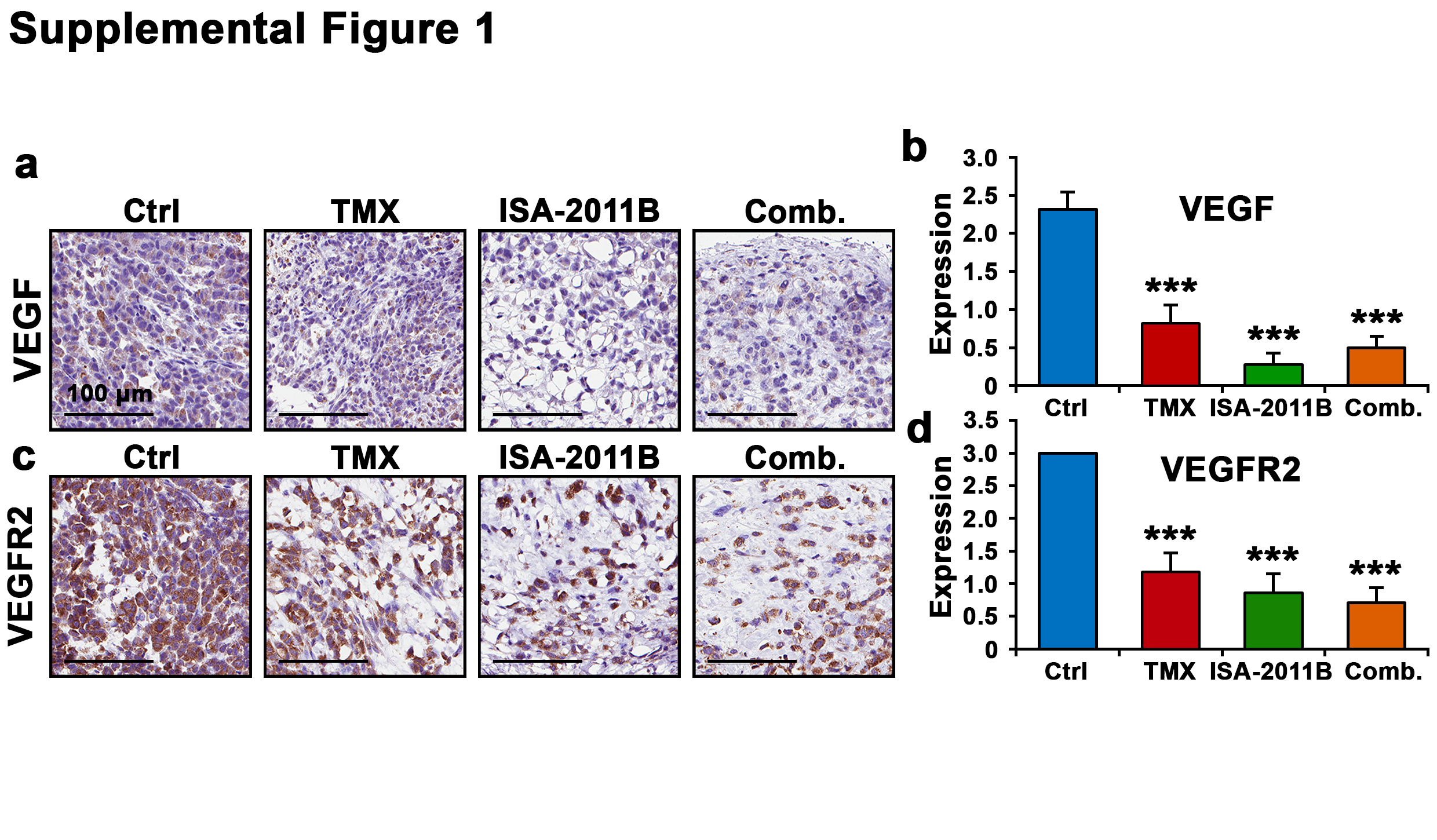

Supplement: Supplementary file 1 — Fig S1. The effect of tamoxifen and ISA‐2011B alone or in combination on VEGF and VEGFR2 expression in PC‐3 xenograft tumors in mice. [file MOL2-15-968-s003.tif]

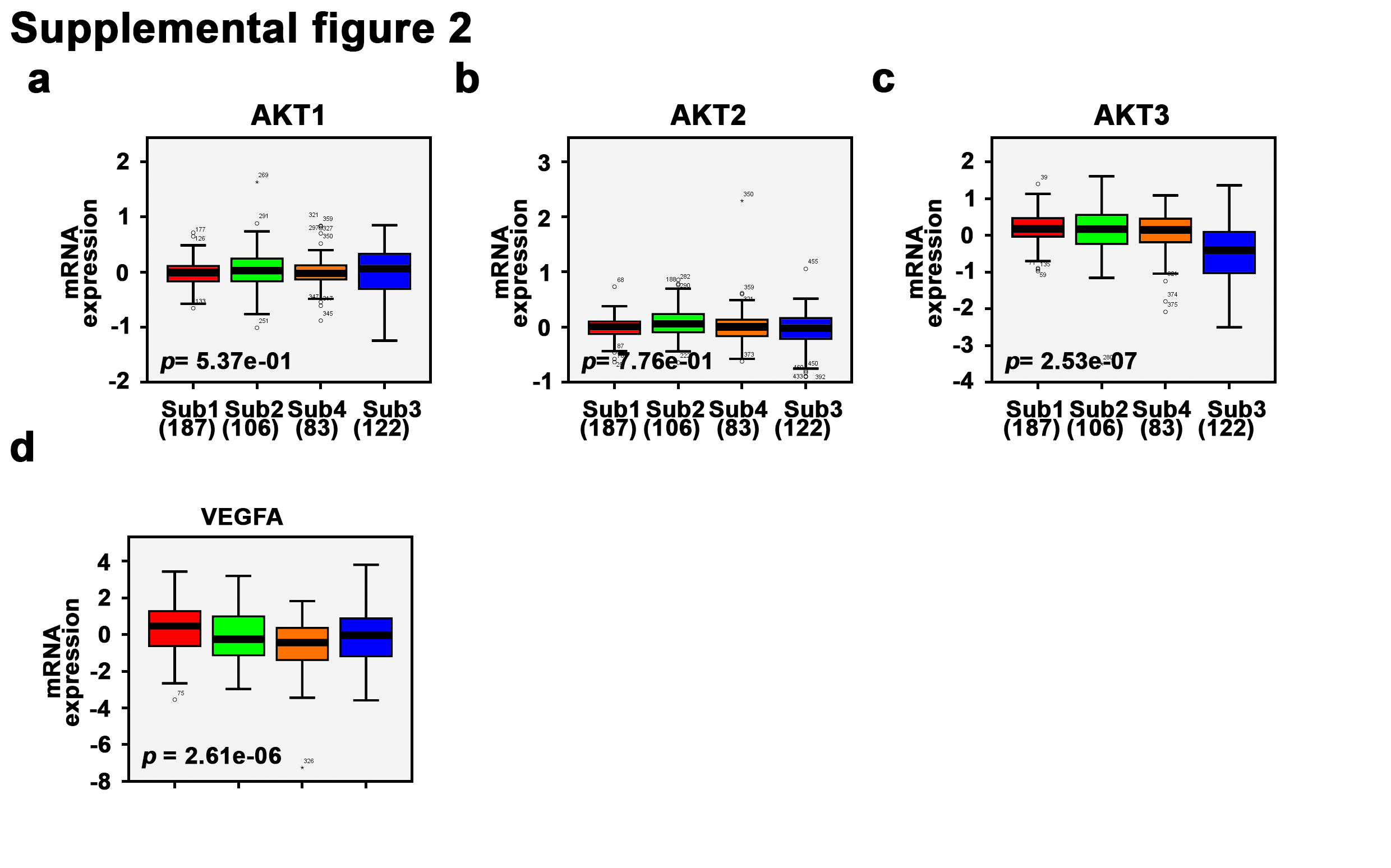

Supplement: Supplementary file 2 — Fig S2. Clinical importance and gene expression signatures of estrogen‐associated signaling pathways in primary cancer tissues from PCa patients. [file MOL2-15-968-s005.tif]

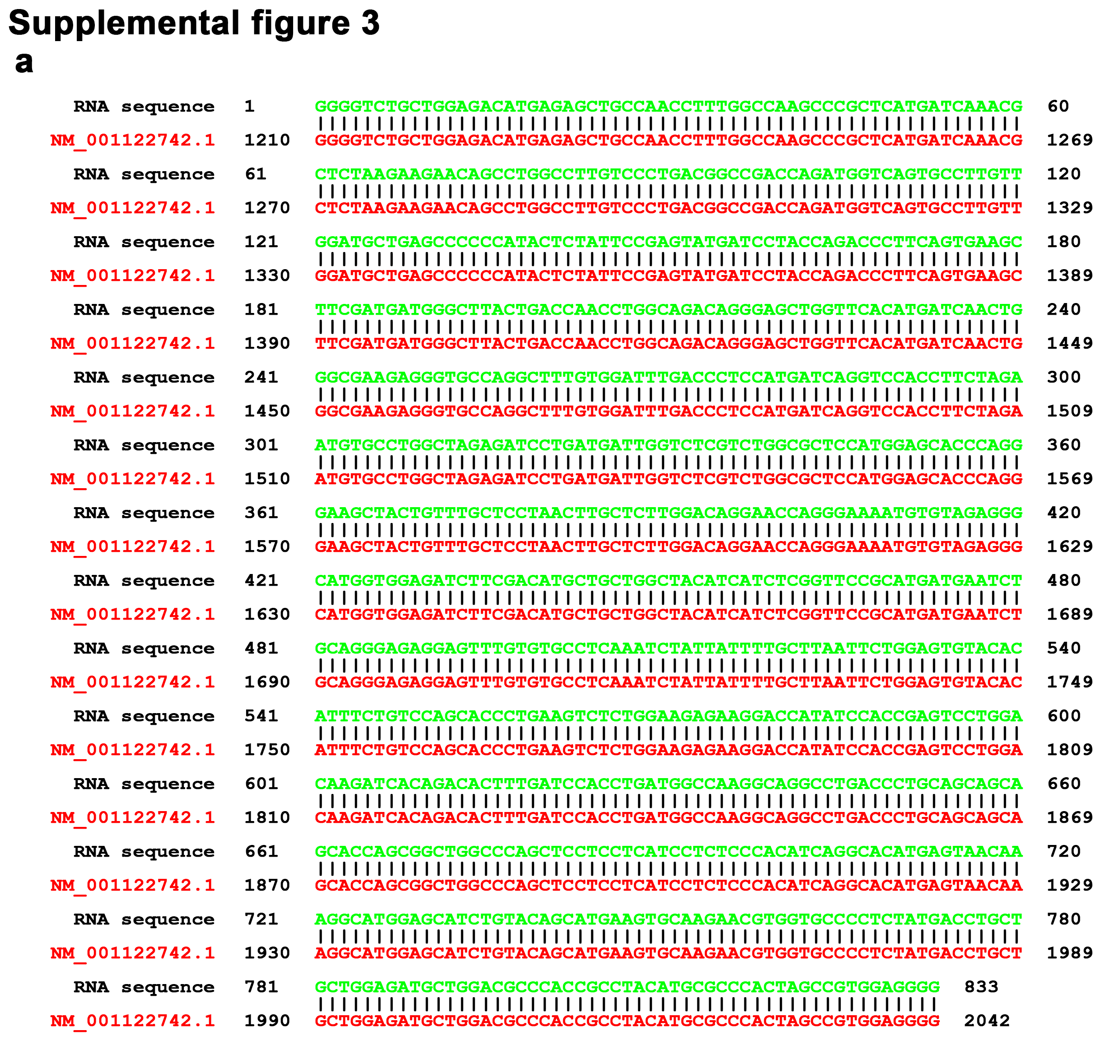

Supplement: Supplementary file 3 — Fig S3. The presence of ESR1 mRNA in PC‐3 cells. [file MOL2-15-968-s004.tif]

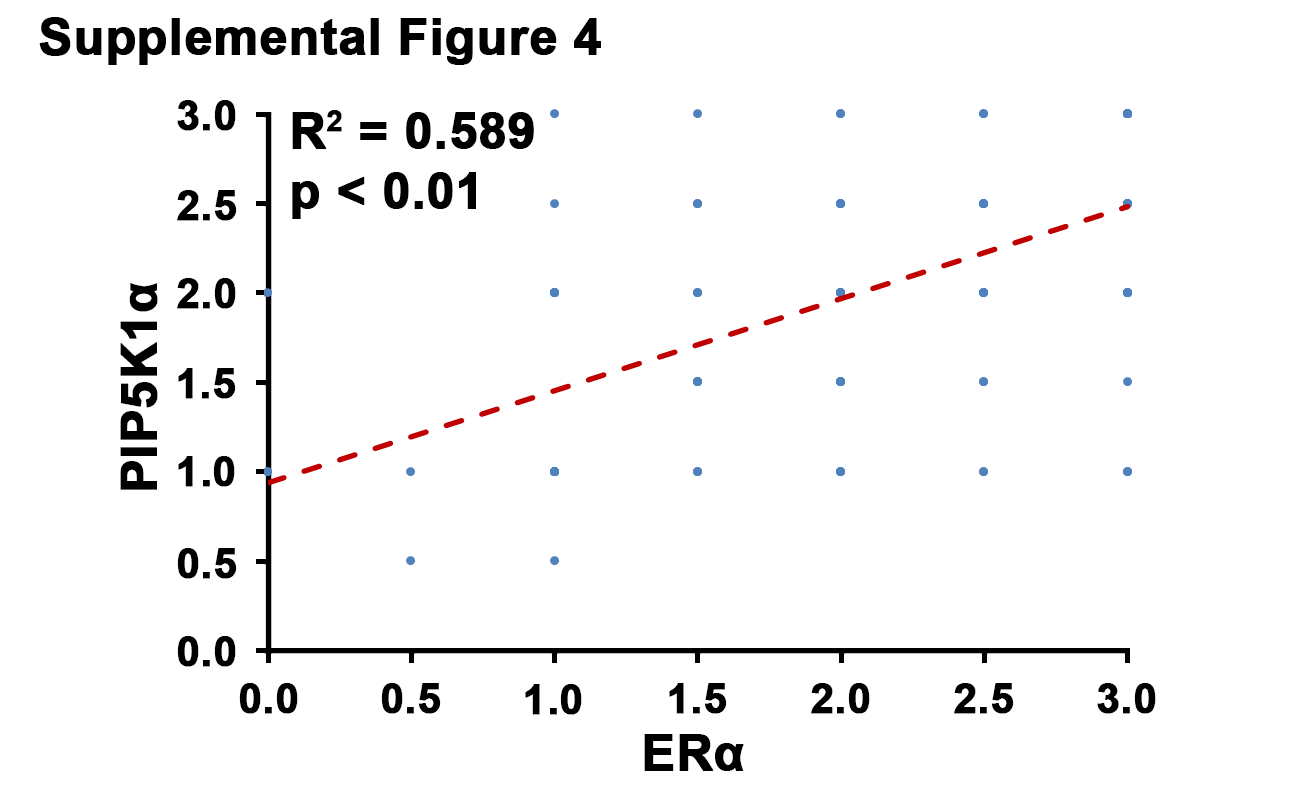

Supplement: Supplementary file 4 — Fig S4. The correlation scatter plot. [file MOL2-15-968-s002.tif]
